# CGsmiles: A Versatile Line Notation for Molecular Representations across Multiple Resolutions

**DOI:** 10.1021/acs.jcim.5c00064

**Published:** 2025-03-24

**Authors:** Fabian Grünewald, Leif Seute, Riccardo Alessandri, Melanie König, Peter C. Kroon

**Affiliations:** † Heidelberg Institute for Theoretical Studies (HITS), Schloss-Wolfsbrunnenweg 35, 69118 Heidelberg, Germany; ‡ Interdisciplinary Center for Scientific Computing, Heidelberg University, 69120 Heidelberg, Germany; § Department of Chemical Engineering, KU Leuven, Celestijnenlaan 200J, 3001 Leuven, Belgium; ∥ Heidelberg University Biochemistry Center, Im Neuenheimer Feld 328, 69120 Heidelberg, Germany; ⊥ Hanze University of Applied Sciences Groningen, Zernikeplein 7, 9747 AS Groningen, The Netherlands

## Abstract

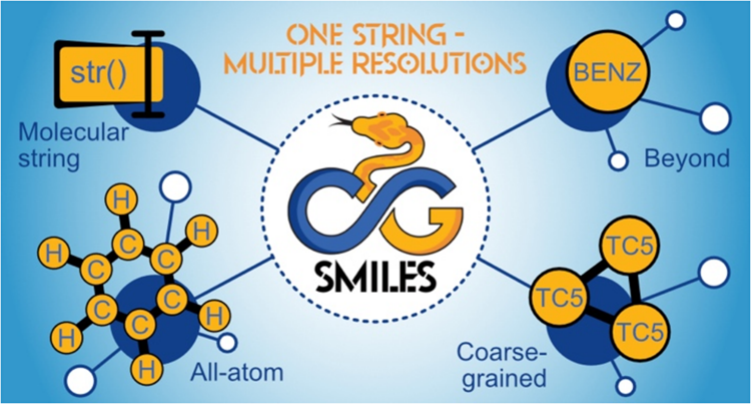

Coarse-grained (CG) models simplify molecular representations by grouping multiple atoms into effective particles, enabling faster simulations and reducing the chemical compound space compared to atomistic methods. Additionally, models with chemical specificity, such as Martini, may extrapolate to cases where experimental data is scarce, making CG methods highly promising for high-throughput (HT) screenings and chemical space exploration. Yet no rigorous data formats exist for the crucial aspect of describing how the atoms are grouped (i.e., the mapping). As CG models advance toward true HT capabilities, the lack of mappings and indexing capabilities for the growing number of CG molecules poses a significant barrier. To address this, we introduce CGsmiles, a versatile line notation inspired by the popular Simplified Molecular Input Line Entry System (SMILES) and BigSMILES. CGsmiles encodes the molecular graph and particle (atom) properties independent of their resolution and incorporates a framework that allows seamless conversion between coarse- and fine-grained resolutions. By specifying fragments that describe how each particle is represented at the next finer resolution (e.g., CG particles to atoms), CGsmiles can represent multiple resolutions and their hierarchical relationships in a single string. In this paper, we present the CGSmiles syntax and analyze a benchmark set of 407 molecules from the Martini force field. We highlight key features missing in existing notations that are essential for accurately describing CG models. To demonstrate the utility of CGsmiles beyond simulations, we construct two simple machine-learning models for predicting partition coefficients, both trained on CGsmiles-indexed data and leveraging information from both CG and atomistic resolutions. Finally, we briefly discuss the applicability of CGsmiles to polymers, which particularly benefit from the multiresolution nature of the notation.

## 1 Introduction

The idea of representing molecules using drawings or string notations is over 250 years old and has since been an integral part of chemistry and the molecular sciences.^1^ With computer sciences entering chemistry and the emergence of chemoinformatics in the 1980s, notations had to become computer-readable and interpretable. To this end, Weininger and co-workers developed the Simplified Molecular-Input Line-Entry System (SMILES), which has been the most popular of such notations ever since.^1,2^ A SMILES string allows researchers to represent a molecule’s connectivity and atomic composition in a single string format. The compact nature of the notation and the fact that it is both human and machine-readable contributed to the success of SMILES. Apart from SMILES, many other notations exist, including for example InChi^3^ and Hierarchical Editing Language for Macromolecules (HELM).^4^

More recently there has been renewed interest in such line notations for their use in chemical language models. A chemical language model trained on a molecule line notation can in principle suggest new chemical compounds by generating a new string representation corresponding to a new molecule. This process can accelerate the exploration of the vast chemical molecular space. In this context, SMILES is frequently criticized because machine learning models trained on them may produce chemically invalid SMILES strings, which do not have a corresponding molecule. To resolve this issue the field has spawned a number of variations and new notation.^5−7^ Among others Krenn et al. have developed Self-Referencing Embedded Strings (SELFIES), which by construction always produce chemically valid molecules.^6^ However, Skinnider recently showed evidence that allowing chemical language models to generate invalid SMILES and removing them only as a postprocessing step can be beneficial. Models that were allowed to produce invalid SMILES showed better chemical space exploration.^8^ In either case, the importance of line notations for machine learning (ML) applications and molecular sciences in general becomes evident.

SMILES and SELFIES, however, quickly become incomprehensible once a molecule exceeds a few hundred atoms. GroupSELFIES^9^ solves this problem by implementing a replacement syntax that allows functional groups to be represented as single units, making the notation more concise. The idea is somewhat similar to the HELM^4^ notation where a hierarchical approach is taken. Instead of specifying each atom in a protein, the sequence can be represented by its amino acid letter codes, each linked to the atomic representation of the amino acid. Such a notation provides a more compact and readable format for large molecules. However, neither approach is sufficient to describe stochastic molecules that lack a well-defined molecular composition such as random copolymers. To address this issue, polymer-specific notations like CurlySMILES^10^ and BigSMILES^11^ have been developed. They capture higher-level structural and atomic information, making them well-suited to create large databases of polymeric molecules. The BigSMILES notation has been augmented to generate new variants, which cover a broader range of chemical contexts, including noncovalently interacting supra polymers for example.^12,13^

To our knowledge, all existing line notations have so far been designed exclusively to represent molecules at atomic level resolution. However, coarse-grained (CG) representations are widely used in molecular modeling.^14−16^ The general idea of any CG model is to group atoms into effective interaction sites, rather than representing each atom individually. This approach reduces the degrees of freedom to be simulated, thereby lowering the computational cost and allowing access to longer time scales. At the same time, by simplifying the molecular representation, CG models can describe multiple similar molecules using the same description, effectively reducing the dimensions of the chemical space.^17^

The origin of CG modeling traces back to simple bead–spring models of polymer melts, where each monomer is represented as one interaction site connected by a spring potential.^18,19^ Such models capture the essential physics of polymers without the need to model the specific chemistry. In contrast, chemically specific coarse-grained (CSCG) models explicitly represent chemically distinct molecules and their interactions. For example, CSCG models of proteins distinguish the individual amino acids to be able to simulate proteins and their residue-specific interactions with lipids, small molecules, and other (bio)molecules.^20^ Transferable CSCG models allow researchers to simulate complex systems with hundreds of chemically distinct molecules and have gained increasing popularity in the fields of material science^15,21^ as well as biophysics.^16,20^ Perhaps the most ambitious application of CSCG modeling to date in terms of complexity and size is the attempt to simulate the entire Syn3A minimal cell using the Martini 3 force field.^22^

To parametrize any CSCG model one needs to know how the atoms of a molecule are grouped, which is commonly referred to as *mapping*. In addition to the grouping, the mapping also describes the quantitative relation between the position of a bead and its constituting atoms. For example, the bead’s position is computed as the mass-weighted average of the atomic positions in a center-of-mass mapping. Mappings are not only essential to reproduce parametrizations but also for tasks like converting coordinates back to the all-atom resolution and performing analysis. While attempts have been made to standardize the mapping procedure,^23^ there is no universally accepted method; mappings depend on the resolution of interest, the force field, and the simulation procedure. Even within a specific simulation approach, there may not exist any well-defined rules, and much less a format for sharing such mappings. The Martini 2 force field is a prime example of this challenge: although it is estimated that the Martini molecule library includes more than three hundred molecules,^24^ mappings are available for only a very small fraction. Even the available mappings typically come in four different formats, which require knowledge of the original molecule coordinate order (Table S1). We hypothesize that as CSCG simulations include more and more molecules and become increasingly complex, the lack of mappings and indexing of CG molecules will become an even more pressing issue.

To address these challenges, we developed the CGsmiles line notation, which can encode molecules at multiple CG resolutions and their conversion between each other as well as to the atomic representation. In addition, a built-in annotation syntax allows the inclusion of additional information such as weights that can describe the quantitative relation between bead and atom positions. The remainder of the paper is structured as follows: We first provide a detailed description of the syntax and the CGsmiles Python API developed around it. Subsequently, we discuss the key features such a notation must support based on a benchmark of about 400 molecules at the Martini 3 force field resolution. Using this database, we illustrate how CGsmiles can be used in ML applications by computing molecular fingerprints and constructing a multiresolution graph neural network to predict Martini partition coefficients. Finally, we explore potential applications in polymer modeling and discuss some limitations as well as future directions for CGsmiles.

## 2 CGsmiles Syntax

The CGsmiles line notation encodes arbitrary resolutions of molecules and defines the conversion between these resolutions unambiguously. Each resolution is explicitly defined and multiple resolutions may be layered together using this notation. At any resolution, a molecule can be expressed as a graph. In this graph, the nodes correspond to (groups of) atoms, such as residues in a protein or polymer, which represent a coarser resolution compared to the next (all-atom) representation. Edges in the graph describe chemical connections between these (groups of) atoms.

With this premise, the first resolution of the CGsmiles notation describes the molecule graph at the coarsest level. Subsequent resolutions define fragments that specify how each node is represented at the next finer resolution (*e.g*. residue to coarse-grained beads, or coarse-grained beads to atoms). Connectivity between nodes at finer resolutions is established through the use of bonding descriptors, which we adapted from the BigSMILES^11^ line notation. Each level of resolution is encapsulated in curly braces and separated by a period. The following sections provide a detailed overview of the syntax underlying the first resolution, the structure of fragments for each of the following resolutions, and some special topics.

### 2.1 Representation of Arbitrary Graphs

The first resolution of the CGsmiles notation captures the coarsest representation of a molecule by adapting the SMILES syntax to represent arbitrary graphs.

#### 2.1.1 Nodes

Nodes within the graph must always be enclosed in square brackets. Inside these brackets, each node is assigned an alphanumeric label preceded by a “#” to distinguish it from standard SMILES notation. While node labels can be arbitrary, they are typically chosen based on the resolution. For example, residue names are used for describing a protein sequence and bead types for a coarse-grained model.

#### 2.1.2 Edges

**Figure 1:**
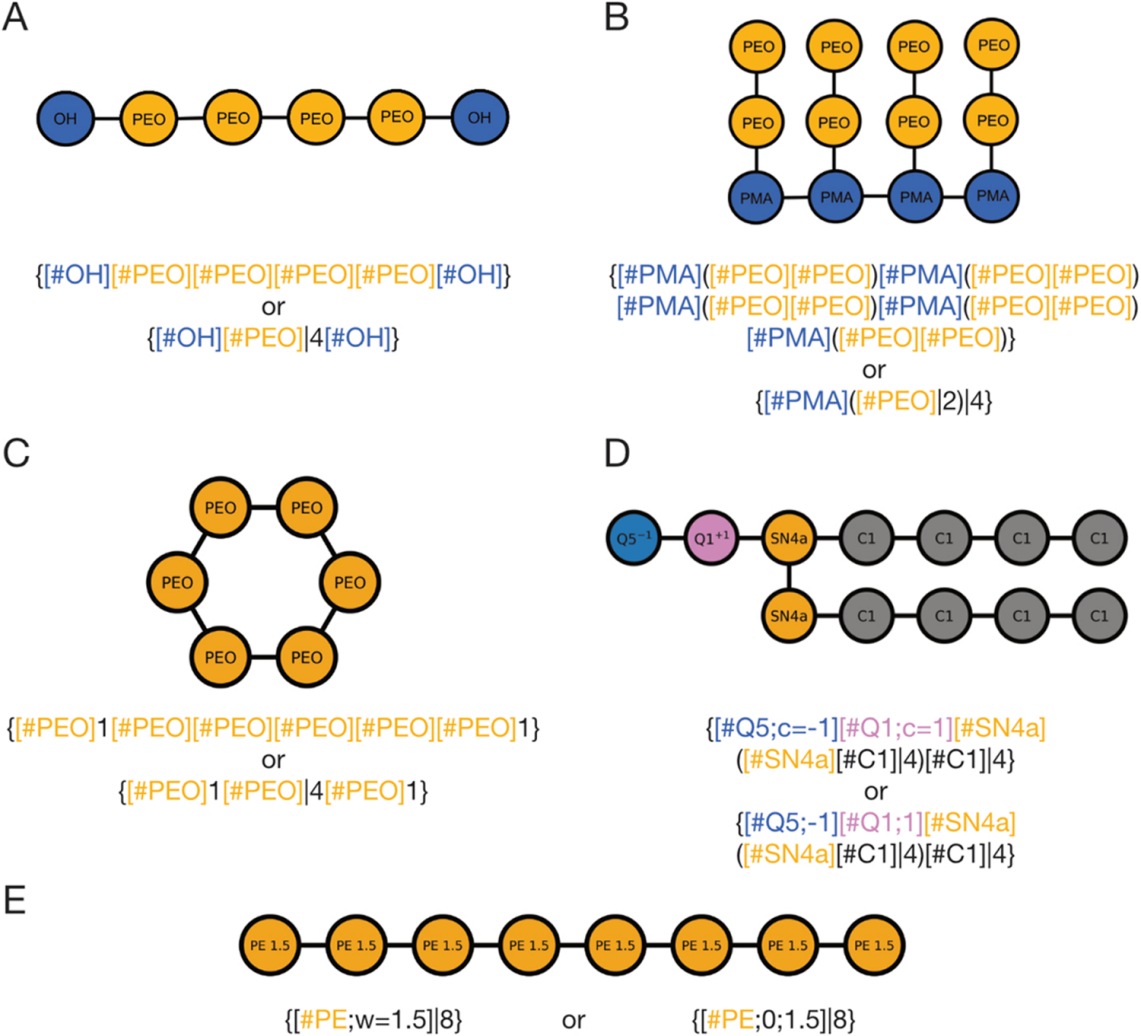
CGsmiles syntax representing example graphs. (A) The linear polymer poly(ethylene glycol) at residue resolution; (B) the polymer brush poly(methyl acrylate-*g*-polyethylene glycol) at residue resolution; (C) the crown ether 18-crown-6 at residue resolution; (D) the lipid dipalmitoylphosphatidylcholine (DPPC) at Martini resolution with charged beads indicated; (E) the linear polymer polyethylene as Kremer-Grest (KG) model, where one node represents 1.5 monomers.

The connections between nodes (i.e., edges) are defined similarly to SMILES. Nodes listed sequentially are interpreted as being linearly connected. For example, Figure 1A illustrates the notation for a poly(ethylene glycol) polymer consisting of four poly(ethylene oxide) (PEO) monomers and two terminal OH groups. For long repetitive sequences, this linear notation becomes cumbersome, therefore, we introduce a multiplication operator “|” to condense the notation. When a multiplication operator follows a node, that node is repeated the specified number of times (see Figure 1A).

#### 2.1.3 Branches

Nonlinear graphs with branches are represented by enclosing each branch in parentheses. The next connection after a closed branch (i.e., after closing parentheses) links to the node preceding the first branching point, consistent with the SMILES notation. If a multiplication operator follows a branch, the entire branch including the branching point is repeated; as shown in Figure 1B, this feature allows for very efficient notation of polymer brushes such as poly(methyl acrylate-*g*-polyethylene glycol).

#### 2.1.4 Rings

Rings are represented by appending an integer to a node, serving as a ring marker. Two nonconsecutive nodes with the same ring marker are connected via an edge thus forming a ring. Figure 1C shows the notation for the crown ether 18-crown-6, a cyclic hexamer of PEO. In addition, CGsmiles also supports the “%” notation from SMILES, which enables the use of multidigit integers as ring markers. Unlike the OpenSMILES standard,^25^ a ring marker may be followed by any number of integers. For example, %123 represents ring marker 123 in CGsmiles, whereas in OpenSMILES, it would refer to ring markers 12 and 3. Ring markers can be reused after the corresponding ring has been closed.

#### 2.1.5 Bond Orders

Any node may be followed by one of the bond-order symbols (“.”, “—”, “=”, “#”, “$”) also used in SMILES, representing bond orders from 0 to 4, respectively. Bond orders indicate the number of connections between two nodes at the next finer level of resolution (see Section 2.3).

#### 2.1.6 Annotations

CGsmiles notation supports a flexible annotation syntax that allows users to attach various attributes to nodes as key-value pairs in the format “key = value”. Annotations are separated from the node label and each other by a semicolon. For example, Figure 4D shows the DPPC lipid at the Martini resolution, where two beads are annotated with their respective charges after the fragment label. The CGsmiles base dialect includes two implicit annotation keys: “**q**” for ***charges*** and “**w**” for ***weights***. These attributes can be specified without explicitly naming the key as shown in the second line of Figure 1D. In chemically specific Kremer-Grest (KG) models it is common that a single bead represents a fractional number of monomers.^26^ This relationship may be indicated using a weight annotation at the CG node. For example, Figure 1E shows the notation for polyethylene, where one CG bead represents 1.5 monomers. Note if no charges are present, weights must be explicitly defined using the “w” key or preceded by a 0 to indicate no charge.

### 2.2 Representation of Fragments

After the first resolution, each subsequent resolution in the CGsmiles string is defined using fragments. Each fragment describes a finer-resolution graph that corresponds to a single node from a coarser resolution.

#### 2.2.1 Fragment Graph

The notation for a fragment graph starts with a “#” followed by the label of the coarser-resolution node and an “=” sign. Each fragment name must be unique to ensure unambiguous identification.

For example, consider the PEO polymer from Figure 1A. At the atomic level, the PEO repeat unit is −CH_2_–O–CH_2_–. In CGsmiles notation, this fragment would be represented as “#PEO=COC”. Fragments can be described either using OpenSMILES^25^ syntax, suitable for molecules at atomic resolution, or CGsmiles graph syntax as described before. Toggling between resolutions is done in the API by providing the language to the parser. The CGsmiles graph syntax supports full annotations, whereas the OpenSMILES syntax permits only weight annotations. For example, when describing a water molecule as one bead, one could place the particle exactly on top of the oxygen atom by writing: {#water=[H;w=0][O;w=1][H;w=0]} or {#water=[H;w=0][O][H;w=0]}. By default, each atom is assigned a weight of one.

#### 2.2.2 Bond Operators

**Figure 2:**
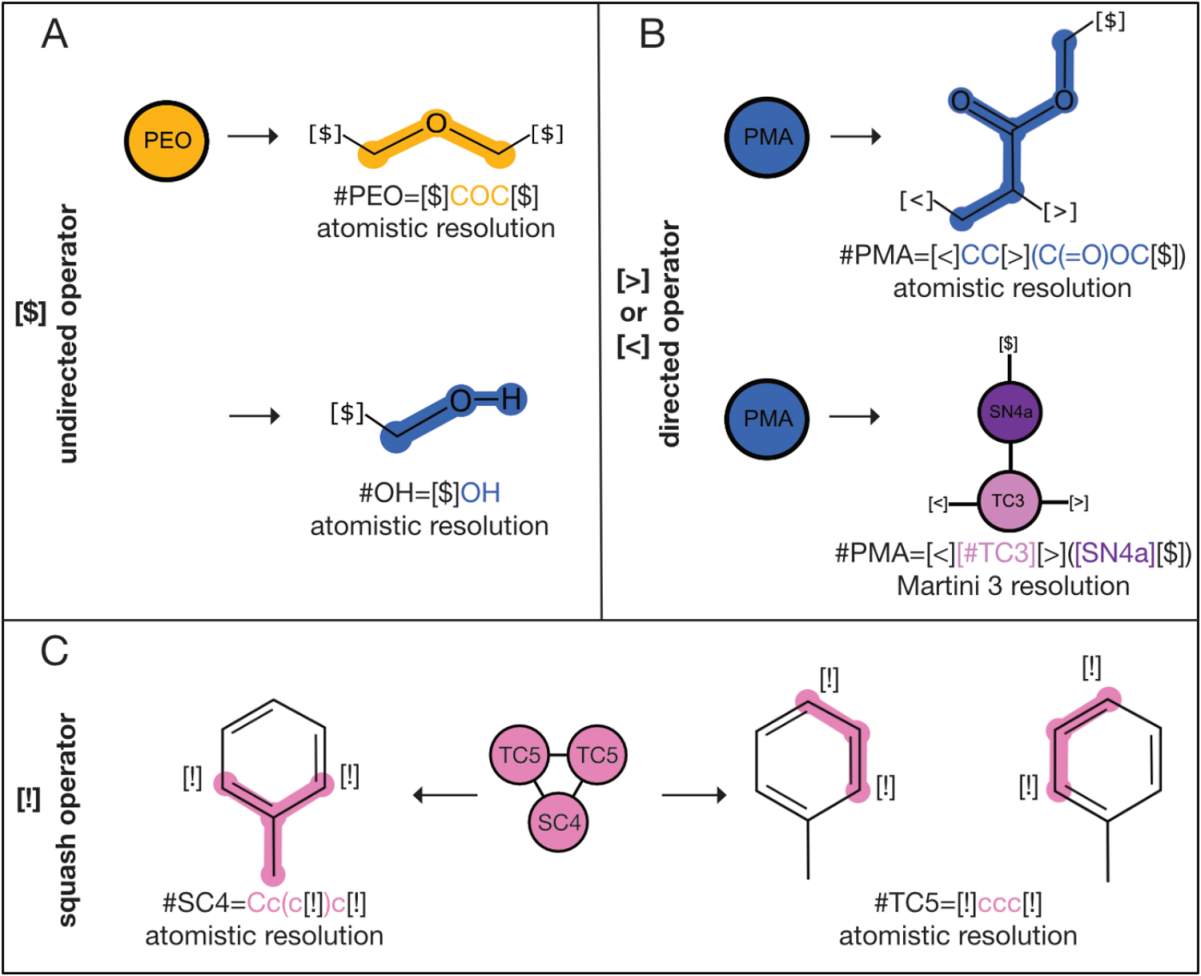
CGsmiles notation for fragments. Fragments describe a single coarse node at a finer resolution either using the general graph notation or OpenSMILES syntax.^25^ In addition, three bonding operators are used to indicate how bonds/edges between fragments are formed. (A) The undirected bonding operator can combine with any other undirected bonding operator to form a bond (e.g., the PEO monomer is symmetric so it may connect on either carbon and the terminal bond only has one operator for connection). (B) The directed operator only combines with its complementary connector that is “>” with “<” or vice versa. In Poly(methyl methacrylate) (PMA) the repeat unit is asymmetric and requires the CH2 carbon to always connect to the CH carbon for head-to-tail addition. At Martini 3 level this symmetry is not present but the operator may still be used for extra emphasis. (C) The squash operator is used to describe scenarios where atoms are shared between coarser nodes. Atoms from different fragments with the same squash operator are considered equivalent and merge at the finer resolution. In the Martini 3 representation of Toluene some carbon atoms of the aromatic ring are split between CG nodes indicated by the squash operator. Placing the atoms of each fragment on top results in the full molecule. All operators may be combined with an alphanumeric label to distinguish nonequivalent operators of the same type.

To define how two consecutive fragments at a finer resolution are connected, CGsmiles builds upon the bonding connector syntax established in BigSMILES to avoid ambiguity.^11^ Any node or atom that connects to a neighboring fragment is followed by one of four bonding connectors (“$”, “>”, “<”, “!”) enclosed in square brackets (Figure 2A–C). In addition, any operator may be combined with an alphanumeric label to distinguish nonequivalent operators of the same type.

##### 2.2.2.1 Undirected Bonding Operator $

The undirected bonding operator “$” connects to any other “$” operator in neighboring fragments, as specified in the coarser resolution graph. For instance, the PEO fragment shown in Figure 2A is symmetric, meaning the order in which the connection is established does not matter. An undirected bonding operator may be followed by an alphanumeric label, ensuring that only operators with matching labels are connected.

##### 2.2.2.2 Directed Bonding Operators > and <

In contrast to the symmetric PEO fragment, the poly(methyl acrylate) (PMA) fragment in Figure 2B is asymmetric, requiring the CH_2_ group to connect to the CH_1_ group in the next residue. To define this connectivity pattern, CGsmiles employs the directed bonding operators “<” and “>”, as used in BigSMILES.^11^ A directed bonding operator can only pair with its complementary counterpart to ensure the correct head-to-tail connectivity in PMA. These bonding operators can also be annotated with an alphanumeric label for further specificity. Using an undirected bonding descriptor in this scenario would result in ambiguity, not distinguishing between combinations of head-to-tail, tail-to-tail, head-to-head, or tail-to-head additions.

##### 2.2.2.3 Shared Bonding Operator !

To address a common scenario in CG force fields where an atom is distributed between two coarser resolution nodes, CGsmiles introduces the shared bonding operator “!”. In the case of toluene represented at the Martini 3 level (Figure 2C) some of the ring atoms are shared between the two CG beads. When two fragments are connected using the shared bonding operator, the atoms at the connection point are merged into a single atom, retaining the bonds from both fragments.

#### 2.2.3 Valency

Unlike BigSMILES, CGsmiles does not enforce valency rules for atoms or nodes, allowing any atom to be followed by multiple bonding operators. In the case of all-atom fragments, the hydrogen count is determined only after the molecule’s full connection is established. Moreover, there is also no distinction between terminal or in-polymer connectors. In cases where a bond of higher order needs to be represented, a bond order symbol should be placed between the node and bonding operator in both fragments. For example, splitting 2-pentene into two fragments results in {[#A][#B]}.{#A=CC=[$],#B=[$]=CCC}, where the bond order symbol “=” indicates a double bond between ethane and propane fragment.

### 2.3 Layering of Resolutions

**Figure 3:**
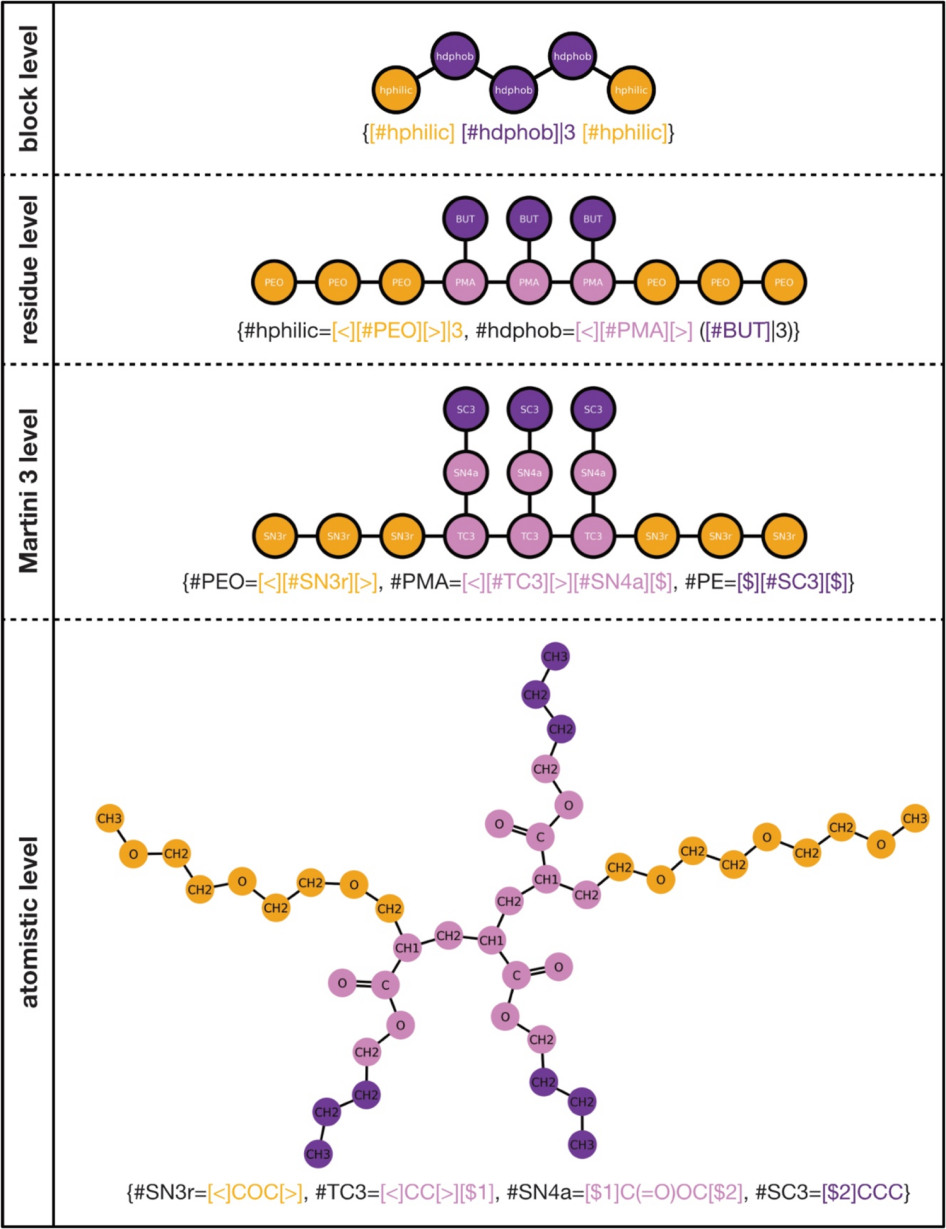
CGsmiles layering of resolutions. The polymer PEG-*co*-PMA-*g*-Butane-*co*-PEG is represented at different levels of resolution (from top to bottom: block, residue, Martini 3, and atomistic levels) and the corresponding CGsmiles are given.

CGsmiles enables the representation of molecular graphs at arbitrary resolutions and their connection to progressively finer resolutions, allowing for the hierarchical layering of multiple levels of details (Figure 3).

#### 2.3.1 Base Graph

The notation starts with the coarsest representation of the system—the base graph. This graph is enclosed in curly braces.

#### 2.3.2 Resolutions

Each additional resolution is represented as a list of fragment graphs, also enclosed in curly braces and separated from the preceding resolution graph by a period. If the final resolution graph is at the atomic level, either CGsmiles or OpenSMILES syntax can be used to describe the fragment graph. This dual approach allows seamless conversion to atomistic resolution using established standards, while also supporting intermediate coarse-grained representations. For example, the methacrylate residue from the branched polymer in Figure 1B can be written at the Martini 3 force field level or at the atomistic level as shown in Figure 2B.

Figure 3 demonstrates this hierarchical layering. A polymer is initially described at the coarsest level with generic labels, such as “hydrophilic” and “hydrophobic” blocks. At the next finer resolution, these blocks are defined in terms of specific residues. Subsequently, the residues are further resolved to the Martini 3 force field description, and finally, the Martini 3 beads are refined to the atomistic level.

#### 2.3.3 Linearizing Rings

**Figure 4:**
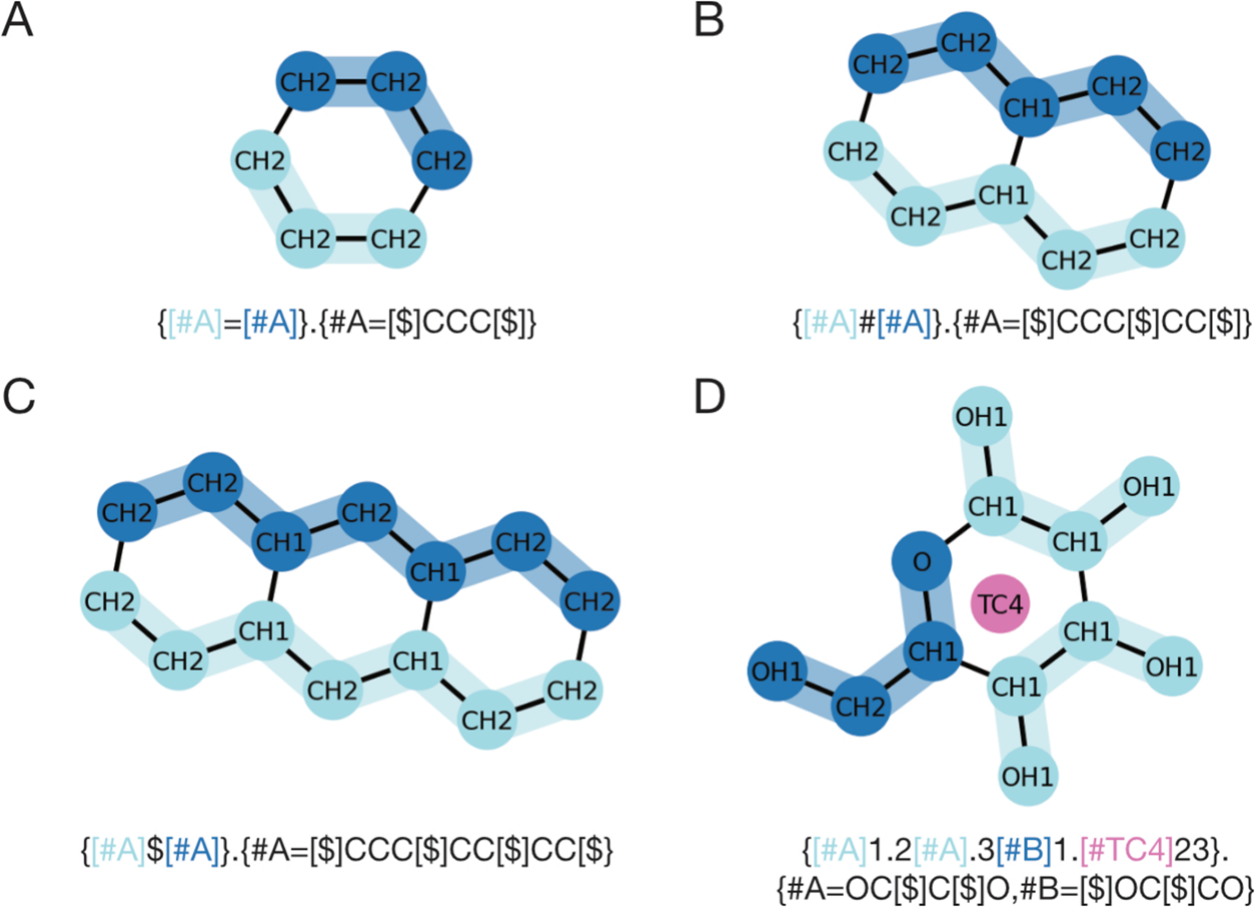
Bond orders in CGsmiles. Bond orders can be used in the CGsmiles graph syntax for cases where the projection of the finer resolution has a different number of bonds than the coarser graph. (A) Cyclohexane at Martini 3 resolution is split into two particles.^27^ While there is one bond at the CG level, the atomic resolution has two bonds. Thus, the bond order is increased to two. (B) When two fused rings are split into two particles one goes from three bonds to one bond. Hence the bond order is increased to three. (C) For three fused rings four bonds are being projected to one at the CG level and thus the bond order becomes four. (D) Zero bond orders can be used to indicate that there is no underlying connection at the finer resolution. For example, in Martini 3 Glucose there is a virtual particle (TC4), which has no corresponding atomic representation but simply aids in the description of the sugar. Zero bond orders are used to signify that this particle is not present in the finer atomic resolution.

As shown in Figure 4, rings at the atomistic resolution can often be mapped into linear structures at the CG level, a common practice in chemically specific force fields such as Martini. In the CGsmiles notation, bond orders at the coarser resolution are utilized to describe such a case. For example, cyclohexane shown in Figure 4A is represented at the Martini 3 level^27^ with a bond order of 2. This order indicates that at the next finer resolution level, two bonds must connect the atoms corresponding to the two CG nodes. This approach also extends to more complex cases, such as splitting fused rings with three or more shared bonds at the CG level. Each additional ring increases the bond order as illustrated in Figure 4B,C. The current CGSimles syntax supports bond orders up to 4, which defines the maximum number of ring connections that can be represented linearly.

#### 2.3.4 Virtual Edges

In certain scenarios, a CG model might include interacting particles that do not correspond to any finer-resolution nodes or atoms. For example, at the Martini 3 resolution glucose, shown in Figure 4D, is represented by three CG particles splitting the sugar ring and one additional virtual particle. The TC4 bead captures the hydrophobic interactions at the ring center but lacks any corresponding fragments at finer resolution.^28^ To accommodate such particles, the CGsmiles notation employs zero bond order edges, referred to as *virtual edges*. Virtual edges are ignored when establishing connections and any particle with only virtual edges is excluded entirely when transitioning to finer resolutions. We note that these virtual edges and virtual particles are not to be confused with the GROMACS virtual sites.^29^ A virtual site in GROMACS describes how a particle’s coordinates are constructed. If a virtual side describes real atoms or CG particles they would be treated as regular nodes rather than virtual ones.

#### 2.3.5 Overloading Wildcards

In certain cases, a single CG graph might describe more than one molecule at the fine-grained resolution because of a loss in resolution at the CG level. An example are Martini lipids such as POPC. POPC can describe lipids with a tail length of 16 or 18 carbons and thus represents at least four molecules when accounting for the position for the double bond. To capture this feature CGsmiles allows to overload the wildcard (*) syntax using annotations. In OpenSMILES^25^ a wildcard means any atom can be placed at the wildcard position. To specify a selection of atoms CGsmiles allows to annotate a wildcard using the *select* keyword abbreviated as “*s*”. Thus, a tail bead in POPC could be written as C1=CCCC[*;s=C,0][*;s=C,0].

### 2.4 Chirality, Isomerism, and Aromaticity

When transitioning between CG and atomistic representations, certain atomistic features have no direct counterparts in CG models and require special treatment.

#### 2.4.1 Implicit Hydrogen

The simplest case is the treatment of implicit hydrogen atoms. SMILES allows for shorthand notation where hydrogen atoms can be omitted and CGsmiles adopts this approach. Hydrogen atoms are automatically assigned once the full atomistic molecule is resolved. This procedure ensures proper handling of any unconsumed bonding operators, which are interpreted as additional hydrogen atoms where applicable. However, hydrogen atoms requiring specific annotations, such as a weight (e.g., “[H;w=0.5]”) must be explicitly included.

#### 2.4.2 Chirality

**Figure 5:**
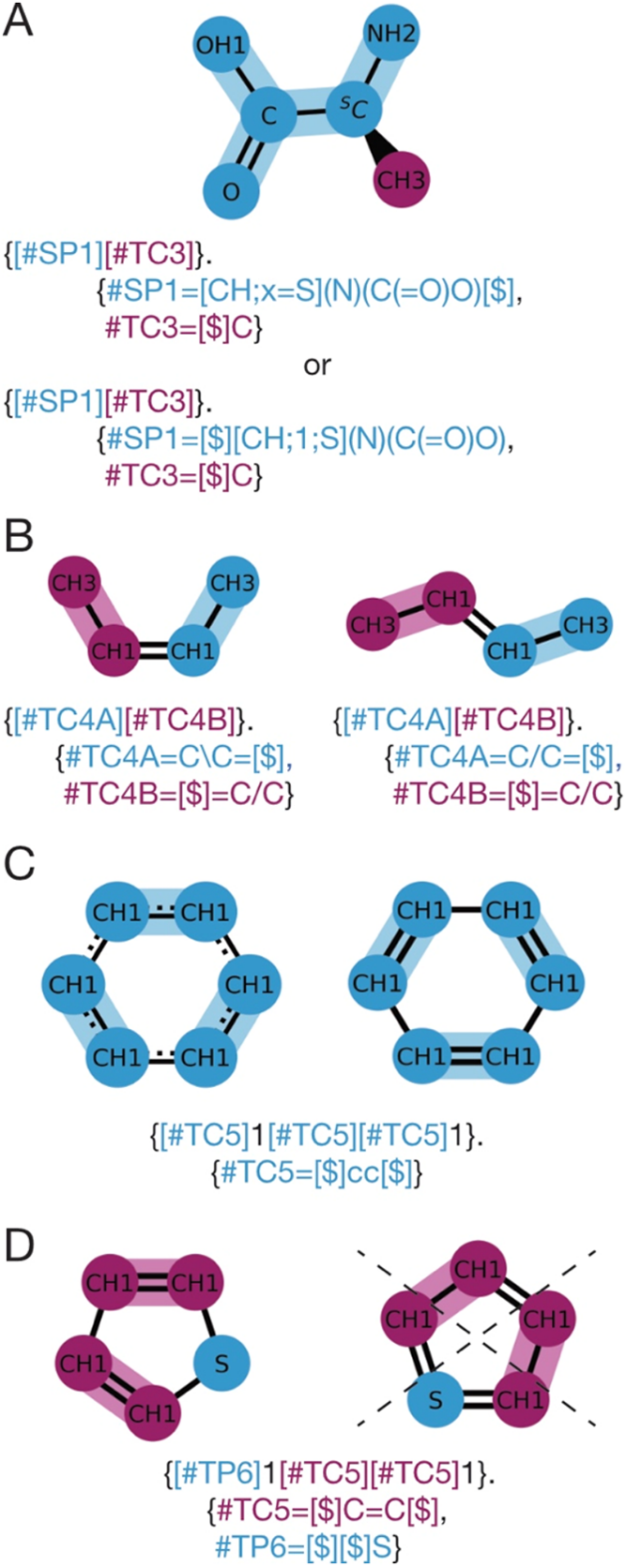
Representation of chirality, isomerism, and aromaticity in CGsmiles. (A) Martini 3 mapping of S-Alanine. The chirality is annotated as using S or R with the keyword “*x*” or as positional argument at second position (i.e., after the weight). (B) Butene has two isomers (*cis* or *trans*), which are represented in CGsmiles using the same symbols (/ \) as in SMILES. Isomers can also be defined when split between two fragments. (C) Two equivalent resonance structures of benzene showing delocalization-induced molecular equivalence (DIME) and the corresponding CGsmiles string. (D) Valid and invalid resonance structure of thiophene. As thiophene only has one valid resonance structure, it does not show DIME and is not aromatic. The correct CGsmiles features double bonds instead of aromatic shorthand.

The SMILES notation uses a local definition of chirality, which has two drawbacks: (1) the chirality depends on the order in which the substituents of the chiral center are listed in the string, and (2) it is not always possible to obtain the absolute configuration from this notation. These issues are further compounded when the order of substituents is determined by the sequence in which fragments are connected, making this approach impractical for CGsmiles. To address these challenges, CGsmiles adopts a more explicit method of chirality assignment using annotations. A chiral atom can be annotated using the “x” keyword as shorthand for chirality. For example, S-Alanine is represented as ′C[C;x=S]C(=O)ON′, while R-Alanine is written as ′C[C;x=R]C(=O)ON′. The x may be omitted if a weight is defined beforehand, such as in ′C[C;1;S]C(=O)ON′, which is also valid (Figure 5A).

#### 2.4.3 *cis*/*trans* Isomerism

*cis* and *trans* isomers are distinguished using a “/” or “\” between atoms to indicate their relative orientation around a double bond, following the OpenSMILES definition. A pair of these symbols defines the isomerism of the two atoms as outlined in Table S2. We note that this notation is permutation invariant, i.e. when double bond substituents are split across fragments, the relative position needs to be assigned only once as if constructing the complete SMILES string (Figure 5B).

#### 2.4.4 Aromaticity

The last concept that requires consideration is aromaticity. In SMILES, aromaticity is encoded using lowercase letters as a shorthand for aromatic atoms or a colon as a marker for aromatic bonds. CGsmiles utilizes the same convention. In addition, aromatic systems may also be split across multiple fragments by simply keeping the shorthand (Figure 5C). For example, Martini benzene is represented as
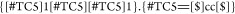


Although the shorthand for aromaticity is well-defined, its interpretation in SMILES remains somewhat ambiguous. To ensure unambiguous valence assignment, necessary for tasks like adding hydrogen atoms, CGsmiles employs the following definition: only atoms capable of participating in delocalization-induced molecular equivalence (i.e., systems where multiple resonance structures can be drawn without introducing charges) are considered aromatic. By this definition benzene is aromatic but thiophene is not (Figure 5D). CGsmiles uses the same definition as Pysmiles package,^30^ which provides a more detailed discussion of this topic. To enhance user-friendliness, the CGsmiles API automatically corrects strings with incorrectly assigned aromaticity at the time of reading. If corrections cannot be made unambiguously, an error is raised, ensuring robust and accurate handling of aromaticity.

### 2.5 Application Programming Interface

To enable seamless use of the CGsmiles line notation, we have developed the CGsmiles Python API, which provides tools for reading, writing, visualizing, and interpreting the notation. A detailed description of the functionality can be found in the documentation https://cgsmiles.readthedocs.io/en/latest/index.html. The CGsmiles package is entirely Python based and depends on the well-established Python packages Numpy,^31^ SciPy,^32^ and NetworkX.^33^ For parsing and interpreting the SMILES-based syntax, the API integrates with the pysmiles package.^30^

The core functionality of the CGsmiles API centers around the *MoleculeResolver* class. This class is initiated from a CGsmiles string and resolves the molecule at its different levels of resolution. For each resolution, two graphs are generated, one representing the molecule at the preceding resolution and another at the current resolution. For maximum interoperability, these graphs are stored as NetworkX graph^33^ objects with node and edge attributes collecting information such as node labels, charges, weights, or any information described in the previous sections. Furthermore, the package provides functionality for writing CGsmiles strings and creating visualizations, like the figures in this manuscript.

## 3 Results and Discussion

String notations for describing molecules at the atomic resolution have a clear number of required features (e.g., branches, rings, or isomerism). Even though extensions have been made to include for example noncovalent interactions,^13^ the core features remain the same across all notations.^4−7,9−12^ On the other hand, for a notation representing coarse resolutions, there is no such standard. The requirements may even vary depending on the CG model. To establish some requirements of features for such a notation, we will showcase three possible applications for CGsmiles.

### 3.1 Library of Martini 3 Mappings

In order to benchmark the CGsmiles notation and collect required features, we compiled a library of 407 CGsmiles strings of unique molecules (Supporting Information) represented in the Martini 3 force field.^27,28,34−37^ The Martini coarse-grained force field is a chemically specific CG force field used for molecular dynamics simulations. The force field is calibrated by matching against a large database of experimentally measured molecular properties. While many CSCG force fields focus on a particular class of molecules (e.g., proteins) the Martini 3 force field can be used for a broad range of molecules from proteins, over lipids, to synthetic polymers and small molecules. This broad applicability makes it an ideally suited benchmark for complexity. As the syntax is general, other CSCG force fields^35,38−42^ should be able to utilize CGsmiles in the same way even though their molecule coverage at the moment is less than that of Martini 3.

Typically, Martini molecules contain three types of information that are of interest when describing the resolution and setting up simulations: (1) The mapping describes which atoms form a CG bead; (2) the bead types and charges define how the CG beads interact in the simulation; (3) the bonded interactions represent covalent interactions and define the shape and size. Thus, we have created CGsmiles of the Martini molecules using the following procedure. The node-labels consist of the bead-type. If two bead-types describe different fragments the node-labels are appended with a capital letter. Partial and full charges are annotated on the Martini resolution. Chirality, weights, and charges are annotated on the fragment string. We note that bonded interactions are not captured by the CGsmiles notation.

Current Martini tools^24,43−50^ require at least three different file types to capture the same information. They need a topology file defining the nonbonded interactions and features of the Martini beads such as the charge. A mapping file describes the transformation from the atomic representation to CG level. Since mapping files are usually sensitive to order of the atoms or the atom names, a matching coordinate file or atomistic topology file is also required. With CGsmiles all this information is contained within a single string. For a lipid molecule (POPC) the CGsmiles string is 236 characters. If one only compares the mapping file (∼3000 characters) it is already a 10-fold compression. Even for small molecules such as benzene, the compression is 4-fold. For these reasons, we anticipate that CGsmiles will greatly simplify the creation, sharing, and curation of mapping files for CSCG force fields.

CGsmiles defines eight language features that are not part of any other notation that makes use of a hierarchical or grouping approach such as HELM or GroupSELFIES.^4,9^ We found these language features essential to describe molecules within the Martini model. Table 1 provides an overview of each feature and how often it occurred in the collection of Martini 3 molecules. Whereas some are more rare (*e.g*. virtual edges) others such as shared atoms make up a significant proportion of the library. We note that all features, with the exception of weights, occur in at least 10 molecules, which shows they are commonly required.

**Table 1:**
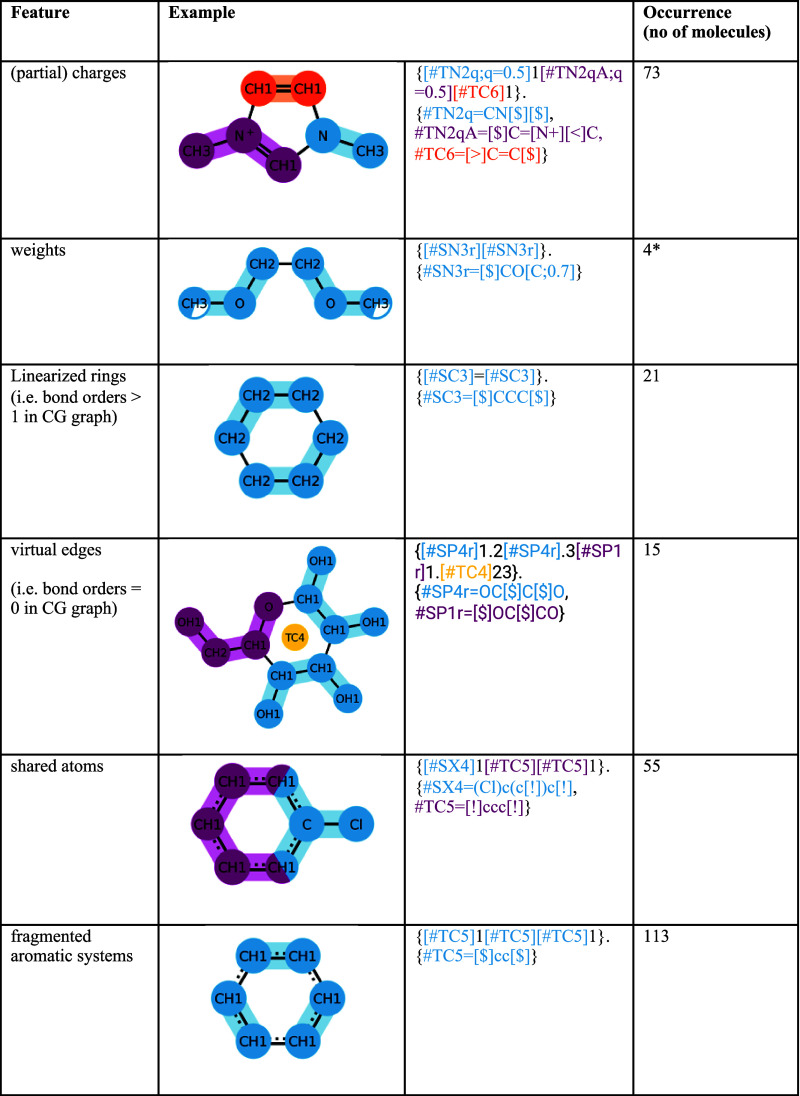
Occurrence of Special Syntax Features in the Benchmark Dataset^a^

a The table lists eight special syntax features required to describe molecules of the Martini 3 force field. The occurrences are counted across the 407 molecules in the benchmark.

* Weights are typically not reported with the mappings.

**Figure 6:**
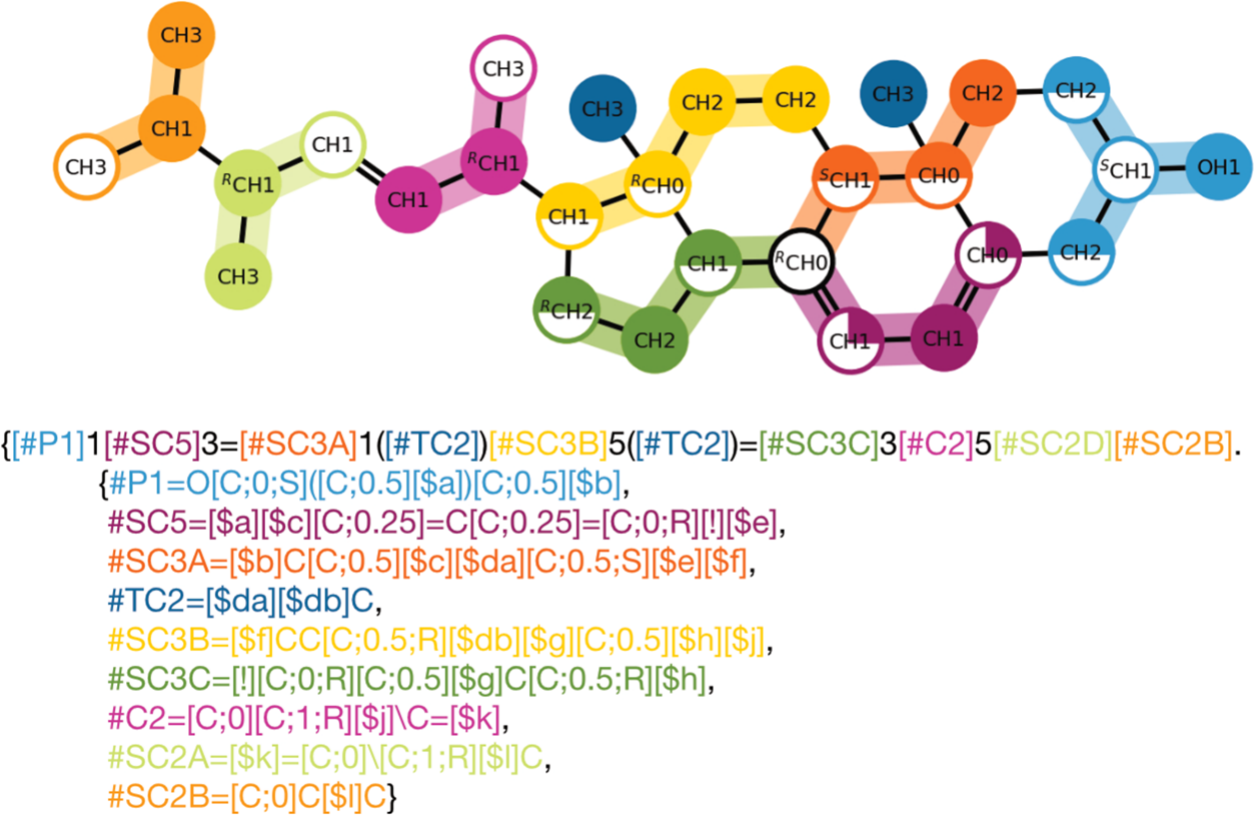
Martini 3 Mapping of Ergosterol. This mapping combines several features (atom sharing, weights, ring-splitting, and *cis*/*trans* isomerism) and is a showcase of the complexity that can emerge at the Martini scale. Hydrogen weights have been omitted for clarity.

There are also molecules that require a combination of these features. One example is Ergosterol (Figure 6). To describe the ergosterol mapping in Martini 3 one requires split rings, *cis*/*trans* isomerism, and weights.^37^ Additionally, Ergosterol has seven chiral centers which can be annotated in the CGsmiles string as indicated by the superscript (R/S). In the current library, the sterols are the most complex examples of molecules. Figure 6 shows the CGsmiles string and corresponding plot.

### 3.2 Machine Learning Applications

Allowing for the representation of complex molecular information in a simple machine-readable string format, line notations are particularly popular for machine learning (ML) applications. Especially when it comes to curating large sets of data from various sources, a standardized cross-compatible format is of great benefit. We hypothesize that CGsmiles can serve a similar purpose when it comes to utilizing the CG representation in ML frameworks. To showcase such applications, we constructed two different ML models to predict the free energy of transfer between water and organic solvents obtained by CG simulations. As a key property used when designing Martini models, the transfer free energy is the largest available set of data for Martini and ideally suited for building a surrogate ML model.

**Figure 7:**
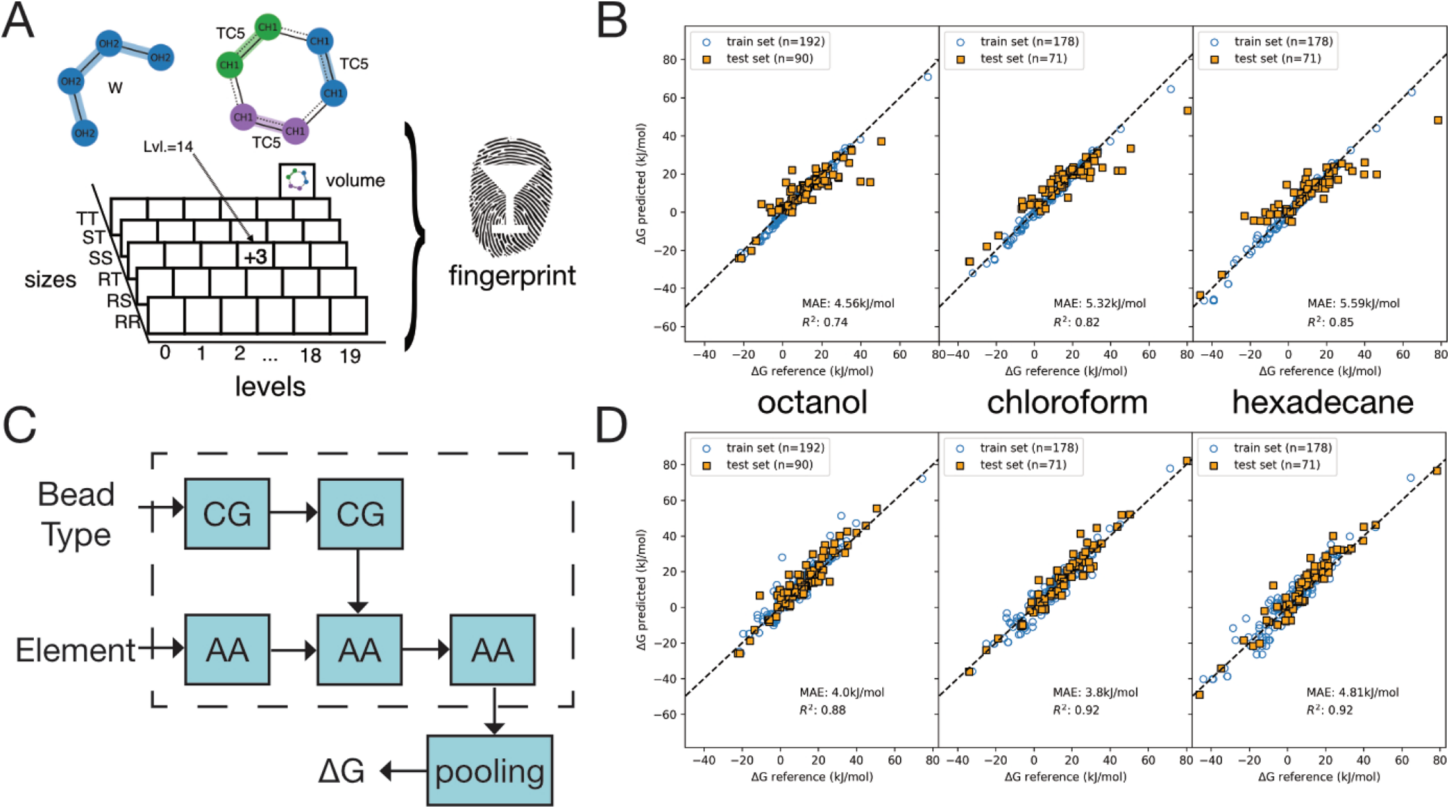
Machine learned prediction of transfer free energies from CGsmiles. We compare the predictions of a random forest with Martini fingerprint (A) for the transfer free energy of unseen solute molecules in (left to right) Octanol, Hexadecane and Chloroform (B) with the predictions of a hierarchical GNN (C, D). The number of target values present in the data set for the respective solvent is denoted by *n*.

Our first strategy is to predict transfer free energies of the Martini 3 model using a random forest with a computed molecular fingerprint that combines CG and all-atom properties (Figure 7A). Martini uses a pairwise definition of interactions between beads. Excluding ionic molecules, these pairwise interactions can have 20 levels going from 0 to 19. Additionally, there are three different bead sizes (regular, small, and tiny) resulting in 6 possible size combinations (regular–regular, regular-small, regular-tiny, small–small, small-tiny, tiny–tiny). In total, this gives rise to 120 types of pairwise interaction, since the interactions are symmetric. Within the Martini force field, the free energy of transfer is determined by the interactions of the solute with water and the solute with an organic solvent. Hence, we construct a fingerprint feature vector by counting how often each interaction type (level and size combination) occurs for all pairs of solute and solvent beads, thus giving rise to a 2 × 120 feature vector. In addition, the molecular volume of the solute is added as a feature and computed from the all-atom graph using RDKits^51,52^ molecular volume function. With this comparatively simple fingerprint as input feature, we train a standard random forest on a set of transfer free energies of 332 solute molecules for the 3 solvents Octanol, Hexadecane, and Chloroform. We randomly split the data set by solute molecule identity, resulting in a training set of 193 molecules, a validation set of 44 molecules for determining hyperparameters, and a set of 99 unseen test solute molecules for assessing the generalization capability of the model. Across the test data set we observe an *R*^2^ of 0.74, 0.82, 0.75 for the three solvents, respectively, and mean-absolute error of 5.1 kJ/mol across all solvents (Figure 7B), indicating that important trends can be captured by the model.

As a second strategy, we propose a graph neural network (GNN)^53^ that operates on both the all-atom and the coarse grained graph, making use of the fact that CGsmiles describe graphs at different resolutions and their interconversion. The GNN updates all-atom and CG node features by performing message-passing with learnable convolutional filters^54^ for each edge type, that is all-atom to all-atom, CG to CG, all-atom to CG and CG to all-atom, where edges between CG and all-atom are defined via fragment membership. We refer to this procedure, which can be readily generalized to more than two resolutions, as *hierarchical message-passing* (Figure 7C). As input features for the CG nodes, we used a one-hot encoding for the size and polarity of the bead; and as input features for the all-atom nodes we used a one-hot encoding for the element and aromaticity, and we encoded the partial charge as scalar. We train the model to predict the transfer free energies for the 3 solvents mentioned above from said input features and the graph connectivity. In order to prevent memorization effects on the data set of less than 300 solute molecules, we only apply three message passing steps; one step on the CG-level followed by one step from CG to all-atom and one step on the all-atom level. We observe that, with an *R*^2^ of 0.88, 0.92, and 0.92 for the three solvents, the GNN achieves overall better performance on the test set of unseen solute molecules (Figure 7D), especially for Hexadecane and Chloroform.

We see another potential use case of our line notation in generative modeling of CG molecules, where a model could be trained to generate CGsmiles strings in analogy to existing approaches for SMILES.^8^ However, the development of such approaches is currently impeded by the lack of large data sets of CG molecules.

### 3.3 Representing Polymers

Aside from representing just two resolutions, CGsmiles is also well suited to represent large polymer molecules with a defined structure. For example, an alternating block copolymer of polyurethane can be written by first representing a linear sequence of 20 PU blocks. In the next level, each PU block is resolved into a PEO block and a urethane linker. The third level defines the atomic structure of these building blocks. For a PU polymer with Toluene diisocyanate as urethane linker and 25 PEO blocks the CGsmiles string is shown below
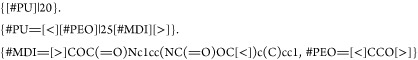


If desired a Martini resolution layer could be inserted. Unlike HELM where each resolution has a specific role (complex polymer, simple polymer, monomer, and atom)^4^ CGsmiles offers arbitrary definition of resolution levels offering a higher flexibility. Compared to the BigSMILES notation, atoms or nodes can have any number of bonding connectors. Thus, their valency is defined by how many bonding connectors are written, and we do not distinguish between terminal and in-line connectors. As terminal fragments have to be explicitly part of the coarse resolution, the notation does not have to account for a separate terminal bonding connector. This convention allows concise writing of hyperbranched molecules. For example, branched polyethylene (PE) can easily be written as shown using a single fragment {#PE=[$]CC[$][$]}.

On the other hand, many polymers have no well-defined molecular structure and are inherently stochastic. Typically, they exist as a distribution over molecular weights and can even be statistical combinations of different monomeric repeat units without a particular sequence. Such molecules are generally poorly described by line notations. For that reason, the BigSMILES^11^ notation was developed to represent both the statistical nature of polymers and the well-defined molecular structure of the monomeric repeat units. Since CGsmiles adopts the bonding connector syntax from BigSMILES, it is also capable of describing a simple statistical copolymer. For example, a truly random copolymer of PS and PMMA can be written as a simple list of fragments omitting the lowest resolution graph
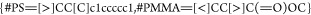


Except for the assignment of monomer names and missing terminal bonding connectors, this string is equivalent to a single statistical object in the BigSMILES notation. These simple statistical polymers can be resolved to defined molecules using the MoleculeSampler class that also allows to fine-tune the composition. Yet more complex statistical polymers, which for example are block copolymers of these simple statistical copolymers, are beyond the scope of CGsmiles. Such cases are well within BigSMILES’s capabilities.

### 3.4 Limitations and Future Outlook

Using the Martini 3 molecule library as a complexity benchmark, we have shown that the CGsmiles language has all features required to describe even very complex mappings. Building on top of this library we were able to utilize the multiresolution nature of this notation to train machine learning models, showcasing CGsmiles’s potential beyond molecular simulations. Additionally, we briefly argued the applicability in the field of polymer science. In this section, we aim to discuss some limitations and possible future applications.

In certain cases, the CGsmiles notation can be sensitive to the ordering of bonding connectors and nodes. Bonding operators are matched along the order of edges determined by the coarse graph. Two fragments are connected by selecting the first node of the fragment and iterating over all bonding operators and nodes until a match is found. This strategy, however, is an implementation choice rather than a language feature. In the original data set of about 407 molecules, there were 40 molecules whose CGsmiles string was sensitive to scrambling the order of CG edges.

**Figure 8:**
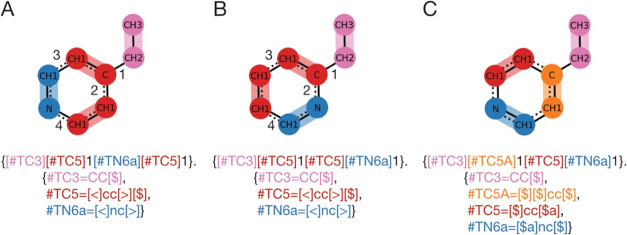
Effect of node permutations on CGsmiles. The figure shows how permutation of nodes in the coarse representation can change the molecule at atomic resolution. The initial definition of 4-ethylpyridine (A) changes to 2-ethylpyridine (B) upon switching the fragment that contains the nitrogen atom with the one containing the aromatic carbons. It is possible to write a permutation insensitive CGsmiles string (C) by defining one additional fragment for the aromatic carbons to which the ethyl group is attached.

Figure 8 provides a concrete example of this ordering sensitivity. The aromatic ring of 2-Ethylpyridine using the Martini 3 representation is split into 3 fragments. Two fragments (TC5, red) represent the same all-atom structure except that one of the fragments connects to the ethyl substituent. For the case in Figure 8A, the all-atom bonds are formed in order as indicated by the numbers in the figure. These orders follow from the string notation of the CG graph (i.e., pink, red, red, blue). The ring bond takes precedence. Since the bond between the red fragments is formed before the ones between red and blue the correct isomer is found (i.e. 4-ethylpyridine). However, in the case of Figure 8B we can see that by changing the order in the CG representation (note that blue and red nodes are exchanged), the other isomer is found (i.e., 2-ethylpyridine). The only way to obtain a truly permutation insensitive CGsmiles is to introduce a new fragment (orange), which enforces that the nitrogen atom cannot be connected to the orange fragment (i.e., orange does not have bonding operator ‘$a’), as shown in Figure 8C.

We note that a correct CGsmiles string will always resolve to the correct molecule once a correct representation is found, as the API preserves edge orders. In addition, the drawing functionality as well as comparison to a reference graph (*e.g*. obtained from SMILES) are easy ways of checking the correctness of one’s representation.

A second, more fundamental, limitation of CGsmiles arises when trying to describe CG graphs whose connectivity does not follow the mapped connectivity of the all-atom graph. For example, the bonded connectivity of Ergosterol (Supporting Figure 1) was designed for numerical stability.^37^ This connectivity does not follow the mapped connectivity pattern of the molecule at the atomistic resolution (see Figure 6). The CGsmiles string, however, still needs to be written following the mapped atomistic connectivity pattern. There is no reliable way to resolve the all-atom molecule from the CG graph defined by the bonded interactions. However, we note that this limitation only applies to a few rather specific Martini molecules.

As discussed in the previous section, CGsmiles currently is limited to simple stochastic polymers. However, the bonding connector syntax defines all information needed to define how repeat units connect even for complex stochastic polymers. The only missing information are the probabilities by which monomers are chosen and the probability of which connectors are used. In the future we will follow the ideas of Generative BigSMILES^11,12^ and annotate these probabilities with the bonding descriptors allowing CGsmiles to define random polymers and resolve them to actual molecules. Such a feature would put CGsmiles on par with (Generative)BigSMILES^11,12^ in terms of the information contained in the string.

The CGsmiles notation was developed to describe different resolutions of molecules making it also useful for multiscale applications. In sequential multiscaling simulation approaches the system is consecutively converted from one resolution to another (e.g., CG to AA). However, aside from sequential multiscaling also concurrent multiscaling approaches are being actively developed.^55^ In concurrent multiscaling approaches, different parts of the system are represented at different resolutions or Hamiltonions in the same simulation. For example, parts may be represented using a classical force field and parts using quantum mechanics. This speeds up simulations while preserving important details in regions where it is needed. CGsmiles could be used to represent such mixed-resolution models either using the annotation syntax or possibly grouping regions with the same Hamaltonion as one coarse-resolution node.

It is the view of the authors that the CGsmiles notation will be useful regardless of the underlying CG modeling framework. However, we expect CGsmiles to be closely integrated with the Martini Ecosystem. The Martini Database (MAD)^24^ is the current effort of the Martini developers to store and make Martini simulation data accessible. CGsmiles would allow automatic API lookups and storing mapping information. Thus, it solves the key problem of frequently missing or order-specific mapping files. Additionally, conversion from all-atom structures becomes more robust as the mapping is independent of the naming or order of the atomic representation. Defining a canonical notation for Martini molecules, which specifies the expected information, such as weights, contained in the CGsmiles string, will likely be a next step.

## 4 Conclusions

For molecular dynamics simulations of large and complex systems, coarse-grained models are frequently used, which group atoms into effective interaction centers called beads, instead of representing them individually. However, this grouping, also referred to as mapping, is not unique and neither exists a good standard for reporting them. In this paper, we have presented the CGsmiles line notation, which can encode multiple consecutive mappings from one representation to another including the atomic resolutions. Furthermore, through the use of annotations to atoms or beads it becomes possible to represent additional information not encoded by the molecular graph such as mapping weights or chirality. To benchmark this notation we collected a library of about 400 mappings at the Martini 3 resolution and compiled CGsmiles strings for all of them. Based on this data set we highlighted eight syntax features unique to CGsmiles, which are essential to successfully describe mappings of coarse-grained models. Aside from showing the applicability of CGsmiles, this data set can serve as a benchmark for other notations in the future. Furthermore, we constructed a Martini fingerprint-based random-forest (RF) model as well as a multiresolution graph neural network (GNN) to predict Martini partition coefficients with the aim of exploring the impact of CGsmiles on machine learning applications. We found that the GNN, which extracts atomic and coarse-grained features from the CGsmiles strings outperforms the RF model. These examples showcase how simpler data collection and curation via CGsmiles can impact ML applications on CG models. Finally, we illustrate how CGsmiles can be used to efficiently describe polymeric molecules and simple statistical polymeric molecules. To conclude, we presented the CGsmiles notation, which combines ideas of SMILES and BigSMILES with a set of new syntax features, to efficiently describe molecules at different resolutions and the interconversion between these resolutions.

## 5 Methods

### 5.1 Martini Molecule Library

To compile the library of Martini 3 molecule mappings we collected mappings, and bead type assignments from the literature.^27,28,34−37^ In addition, 51 new molecules were generated, which were not published before. Bonded interactions for the rigid molecules were designed following the recommendations of the Martini Small Molecules paper.^27^ Parameters for these interactions were obtained by embedding the three-dimensional (3D) geometry using RDKit,^51^ mapping to CG resolution, and subsequently measuring the bond distances. For flexible molecules, angles were measured and the force constant set to a generic value of 50 kJ/mol. Each CGsmiles string contains two resolutions (i.e., Martini 3 and atomic). For the Martini 3 resolution, we chose to label the nodes by bead type. However, occasionally two different fragments are assigned the same bead type in which case the type was appended with a capital letter A–Z. As these letters are not part of the Martini bead type descriptors, the type can easily be recovered. Weights, chirality, and *cis*/*trans* isomerism was annotated whenever it was part of the mapping files or explicitly discussed in the paper.

### 5.2 Free Energies

Free energies of transfer between water and three organic solvents (octanol, chloroform, hexadecane) of the Martini 3 molecules were collected from previously published literature.^27,28,34−36^ In several cases, the free energy value of one or more solvents was missing. Those values were recomputed following the standard procedure outlined in the Martini 3 parametrization paper.^35^ In particular, the solvation free energies were computed using alchemical free energy transformation as implemented in GROMACS 2023.3. The free energy of transfer is then computed as the difference in solvation free energies. Initial coordinates were built using polyply,^44^ an energy minimization was run, and the system was equilibrated using a NpT simulation of 12 ns with Berendsen Pressure (τ_*p*_ = 1 ps) and Temperature Coupling (τ_*t*_ = 4 ps).^56^ In the subsequent stage, 19 non-equally spaced windows were used to switch off the LJ interactions. Since all Martini molecules considered are neutral, Coulomb interactions play no role. Soft-core LJ potentials were applied following the recommended values.^57^ Each window was run under NpT conditions for 12 ns at 1 bar pressure maintained using the Parrinello–Rahman pressure coupling (τ_*p*_ = 4 ps).^58^ The v-rescale algorithm by Bussi et al.^59^ was used to maintain temperature at 298.15 K. The derivative of the potential energy was recorded every 10 steps. All free energies of the transformation were estimated using the Bennetts Acceptance Ratio (BAR)^60^ method as implemented in the “gmx bar” tool. The statistical error estimate for all calculations was less than 0.2 kJ/mol. We note that statistical errors are omitted in the log *P* training data set as they were not of interest in the machine learning test cases.

### 5.3 Learning Partition Free Energies

For learning transfer free energies of a solute in any of the three solvents (Section 3.2), we train both models considered on a set of transfer free energies of a set of solute molecules in all three solvents. Crucially, we hold out test and validation solute molecules completely by splitting the data set by solute identity. We consider this restrictive splitting as more relevant for evaluating usability in practical applications than the common procedure of splitting the data set merely by the combination of solute and solvent. Predicting the transfer free energy in a given solvent might be easier if the transfer free energy in another solvent is already known. Thus, it is a stronger test to evaluate the performance on entirely unseen solute molecules.

### 5.4 Random Forest Model

The Random Forest Model was trained using the SciKitLearn^61^ library. The same training, test, and validation data set was used as for the GNN architecture. Hyperparameters (*n_estimators, max_depth, min_samples_split, max_features*) were optimized using the Optuna package^62^ to minimize the mean absolute error measured using the validation set. The optimization yielded 114, 23, 2, and  as optimal parameters, respectively. In a second step a linear correction was fit to account for a systematic deviation observed in the data set. This linear correction was optimized again using the validation data set. The final free energy was computed as Δ*G =* −0.11 Δ*G*_rf_ – 0.636.

### 5.5 Hierarchical Graph Neural Network Architecture

We implement the hierarchical graph neural network described in Section 3.2 using the DGL library.^63,64^ We first apply a node-wise linear layer with weights shared across nodes of the same node type, followed by an ELU^65^ nonlinearity. An intermediate feature dimension of 64 is used across the layers. For message passing, we use graph convolutional filters^54^ with weights that are shared across edges of the same type. For the model at hand, we use three message passing steps, as described above, and apply a final pooling layer, in which we sum over all-atom node features and normalize by the number of all-atom nodes exponentiated by a learnable number, which we constrain to lie between zero and one. During training, we minimize the mean squared error between this pooled feature and the target transfer free energy values.

## Data Availability

The source code of the reference implementation is available on GitHub at https://github.com/gruenewald-lab/CGsmiles. The source code and training data for the machine learning models is available on GitHub at https://github.com/LeifSeute/log_p_gnn. Documentation and tutorials for CGsmiles are available online https://cgsmiles.readthedocs.io/en/latest/index.html. Molecular Dynamics data including starting conformation, topology files, and trajectories are available on Zenodo 10.5281/zenodo.14652719.

## Supporting Information Available

Formats of common Martini mapping files; decision table for assigning *cis**trans* labels in CGsmiles; and mapping and bonded interactions of Martini Ergosterol (PDF)
(XLSX)
