# Supplementary material for: CGsmiles: A Versatile Line Notation for Molecular Representations across Multiple Resolutions
Source: J Chem Inf Model. 2025 Mar 24;65(7):3405–19. doi: 10.1021/acs.jcim.5c00064 (PMC12005186; doi:10.1021/acs.jcim.5c00064)
Supplement: Supplementary file 1 — ci5c00064_si_001.pdf [file ci5c00064_si_001.pdf]

Supporting Information

CGsmiles: A Versatile Line Notation for Molecular Representations Across Multiple Resolutions

Fabian Grünewald[1,2]*, Leif Seute[1], Riccardo Alessandri[3], Melanie König[4], Peter C. Kroon[5]

1 Heidelberg Institute for Theoretical Studies (HITS), Schloss-Wolfsbrunnenweg 35, 69118 Heidelberg, Germany

2 Interdisciplinary Center for Scientific Computing, Heidelberg University, Heidelberg, Germany

3 Department of Chemical Engineering, KU Leuven, Celestijnenlaan 200J, 3001 Leuven, Belgium

4 Heidelberg University Biochemistry Center, Im Neuenheimer Feld 328, 69120 Heidelberg Germany

5 Hanze University of Applied Sciences Groningen, Zernikeplein 7, 9747 AS Groningen, The Netherlands

* corresponding author

Table S1. Common Formats for Mapping Files in Martini

**Program**	**Extension**	**Description**
Backwards[1]	.map	Requires matching all-atom topology by names and residue names
Vermouth[2]	.mapping	Requires matching all-atom topology by names and residue names
Bartender [3]	.inp	Requires coordinates matching in order
GROMACS[4]	.ndx	Requires coordinates matching in order
PyCGTOOL[5]	.map	Requires matching molecule names and atom names
CG2AT[6]	N/A	Fragment files matching force field topology at the all-atom level

Table S2. Assignment of cis/trans isomerism from SMILES markers following the OpenSMILES guidelines[7]

Category	Example	Assignment
/ / - no branch	F/C=C/F	trans
\ / - no branch	F\C=C/F	cis
/ \ - no branch	F/C=C\F	cis
\ \ - no branch	F\C=C\F	trans
\ / - branch	C(\F)=C/F	trans
/ / - branch	C(/F)=C/F	cis
/ \ - branch	C(/F)=C\F	trans
\ \ - branch	C(\F)=C\F	cis

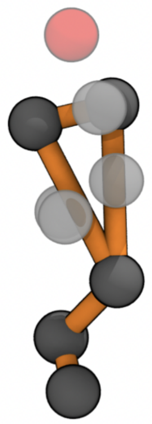

Figure S1 Bonded Connectivity in Martini 3 Ergosterol.[8] Dark gray spheres show the Martini beads which are connected by bonded potentials. Beads shown in light gray and light red are modelled as so-called virtual sites. The position of virtual sites are constructed as linear combinations of the position of the other particles.

References

(1) Wassenaar, T. A.; Pluhackova, K.; Böckmann, R. A.; Marrink, S. J.; Tieleman, D. P. Going Backward: A Flexible Geometric Approach to Reverse Transformation from Coarse Grained to Atomistic Models. *J. Chem. Theory Comput.* **2014**, *10* (2), 676–690. https://doi.org/10.1021/ct400617g.

(2) Kroon, P. C.; Grünewald, F.; Barnoud, J.; van Tilburg, M.; Souza, P. C. T.; Wassenaar, T. A.; Marrink, S.-J. Martinize2 and Vermouth: Unified Framework for Topology Generation. *ArXiv Submitt.* **2022**.

(3) Pereira, G. P.; Alessandri, R.; Domínguez, M.; Araya-Osorio, R.; Grünewald, L.; Borges-Araújo, L.; Wu, S.; Marrink, S. J.; Souza, P. C. T.; Mera-Adasme, R. Bartender: Martini 3 Bonded Terms via Quantum Mechanics-Based Molecular Dynamics. *J. Chem. Theory Comput.* **2024**, *20* (13), 5763–5773. https://doi.org/10.1021/acs.jctc.4c00275.

(4) Abraham, M. J.; Murtola, T.; Schulz, R.; Páll, S.; Smith, J. C.; Hess, B.; Lindah, E. Gromacs: High Performance Molecular Simulations through Multi-Level Parallelism from Laptops to Supercomputers. *SoftwareX* **2015**, *1–2*, 19–25. https://doi.org/10.1016/j.softx.2015.06.001.

(5) Graham, J. A.; Essex, J. W.; Khalid, S. PyCGTOOL: Automated Generation of Coarse-Grained Molecular Dynamics Models from Atomistic Trajectories. *J. Chem. Inf. Model.* **2017**, *57* (4), 650–656. https://doi.org/10.1021/acs.jcim.7b00096.

(6) Vickery, O. N.; Stansfeld, P. J. CG2AT2: An Enhanced Fragment-Based Approach for Serial Multi-Scale Molecular Dynamics Simulations. *J. Chem. Theory Comput.* **2021**, *17* (10), 6472–6482. https://doi.org/10.1021/acs.jctc.1c00295.

(7) James, C. A. OpenSMILES Specification, 2016. https://github.com/opensmiles/OpenSMILES (accessed 2025-03-01).

(8) Kjølbye, L. R.; Valério, M.; Paloncýová, M.; Borges-Araújo, L.; Pestana-Nobles, R.; Grünewald, F.; Bruininks, B. M. H.; Araya-Osorio, R.; Šrejber, M.; Mera-Adasme, R.; Monticell, L.; Marrink, S. J.; Otyepka, M.; Wu, S.; Souza, P. C. T. Martini 3 Building Blocks for Lipid Nanoparticle Design. ChemRxiv September 24, 2024. https://doi.org/10.26434/chemrxiv-2024-bf4n8.
